# Endemic Systemic Mycoses in Italy: A Systematic Review of Literature and a Practical Update

**DOI:** 10.1007/s11046-023-00735-z

**Published:** 2023-06-09

**Authors:** Verena Zerbato, Stefano Di Bella, Riccardo Pol, Francesco D’Aleo, Andrea Angheben, Claudio Farina, Marco Conte, Francesco Luzzaro, Gianluigi Lombardi on behalf of the AMCLI Mycology Committee, Roberto Luzzati, Luigi Principe

**Affiliations:** 1 Infectious Diseases Unit, Trieste University Hospital (ASUGI), Piazza dell’Ospitale 1, 34125 Trieste, Italy; 2 Microbiology and Virology Unit, Great Metropolitan Hospital “Bianchi Melacrino Morelli”, 89124 Reggio Calabria, Italy; 3 Department of Infectious, Tropical Diseases and Microbiology, IRCCS Sacro Cuore - Don Calabria Hospital, 37024 Negrar di Valpolicella, Verona, Italy; 4 Microbiology and Virology Laboratory, ASST “Papa Giovanni XXIII”, 24127 Bergamo, Italy; 5 Clinical Microbiology and Virology Unit, “A. Manzoni” Hospital, 23900 Lecco, Italy; 6 Clinical Microbiology, ASST Grande Ospedale Metropolitano Niguarda, 20121 Milan, Italy; 7 Clinical Department of Medical, Surgical and Health Sciences, Trieste University, 34149 Trieste, Italy; 8 Clinical Pathology and Microbiology Unit, “S. Giovanni di Dio” Hospital, 88900 Crotone, Italy

**Keywords:** Systemic mycoses, Talaromycosis, Paracoccidioidomycosis, Blastomycosis, Coccidioidomycosis, Histoplasmosis

## Abstract

Endemic systemic mycoses such as blastomycosis, coccidioidomycosis, histoplasmosis, talaromycosis, paracoccidioidomycosis are emerging as an important cause of morbidity and mortality worldwide. We conducted a systematic review on endemic systemic mycoses reported in Italy from 1914 to nowadays. We found out: 105 cases of histoplasmosis, 15 of paracoccidioidomycosis, 10 of coccidioidomycosis, 10 of blastomycosis and 3 of talaromycosis. Most cases have been reported in returning travelers and expatriates or immigrants. Thirtytwo patients did not have a story of traveling to an endemic area. Fortysix subjects had HIV/AIDS. Immunosuppression was the major risk factor for getting these infections and for severe outcomes. We provided an overview on microbiological characteristics and clinical management principles of systemic endemic mycoses with a focus on the cases reported in Italy.

**Supplementary Information:**

The online version contains supplementary material available at 10.1007/s11046-023-00735-z.

## Introduction

The most common endemic systemic mycoses are caused by thermally dimorphic fungi [1]. This group of fungi grows as a mold at 22–25 °C and as yeasts at 37 °C [2]. Among these diseases, blastomycosis, coccidioidomycosis, histoplasmosis, talaromycosis, paracoccidioidomycosis are emerging as an important cause of morbidity and mortality worldwide [3]. They have a peculiar geographical distribution in most cases. Migratory flows and travels allow their spread also in non-endemic countries [4]. They can affect both immunocompetent and immunocompromised people, particularly those with HIV/AIDS, where they manifest with more severe outcomes [5]. Recently the International Society for Human and Animal Mycology and the European Confederation of Medical Mycology published guidelines for the diagnosis and management of the endemic mycoses [3].

In Italy many case reports and case series have been published about endemic mycoses, mostly imported from endemic areas. Systematic reviews regarding Italian cases have been conducted only about histoplasmosis [4, 6–8]. A review about cases of paracoccidioidomycosis in Europe was conducted by Wagner et al. [9].

We conducted a systematic review on endemic systemic mycoses reported in Italy. A practical update of microbiological and clinical aspects is provided.

## Materials and Methods

This systematic review was performed according to the Preferred Reporting Items for Systematic reviews and Meta-Analyses (PRISMA) [10].

Information sources were represented by three major databases, MEDLINE, CENTRAL and Embase, screened from inception until 1st April 2022 using a combination of keywords. The detailed search strategy is described in Appendix 1 (See supplementary materials).

Records were de-duplicated before entering the subsequent phase of the review. One investigator (VZ) carried out the first selection of the retrieved records by title and abstract in order to establish eligibility for full-text review. The second step consisted of further screening of full-text articles to define final inclusion in the systematic review according to the inclusion criteria. We included case reports, case series, systematic reviews about the following mycoses: histoplasmosis, coccidioidomycosis, talaromycosis, blastomycosis, and paracoccidioidomycosis. We included full texts written in English and Italian. Additional cases were sought from the reference list of included papers and reviews.

The following information was extracted from each article and entered into pilot-tested evidence tables: mycosis, author, year, number of cases, patients’ nationality, age, gender, comorbidities, immunocompromised status, country of exposure, clinical presentation and affected organs, isolated species, co-infections, diagnosis, antifungal therapy, and outcomes.

The authors confirm that the ethical policies of the journal, as noted on the journal’s author guidelines page, have been adhered to. No ethical approval was required as this is a review article with no original research data.

## Results

### Summary of the Literature

**Fig. 1:**
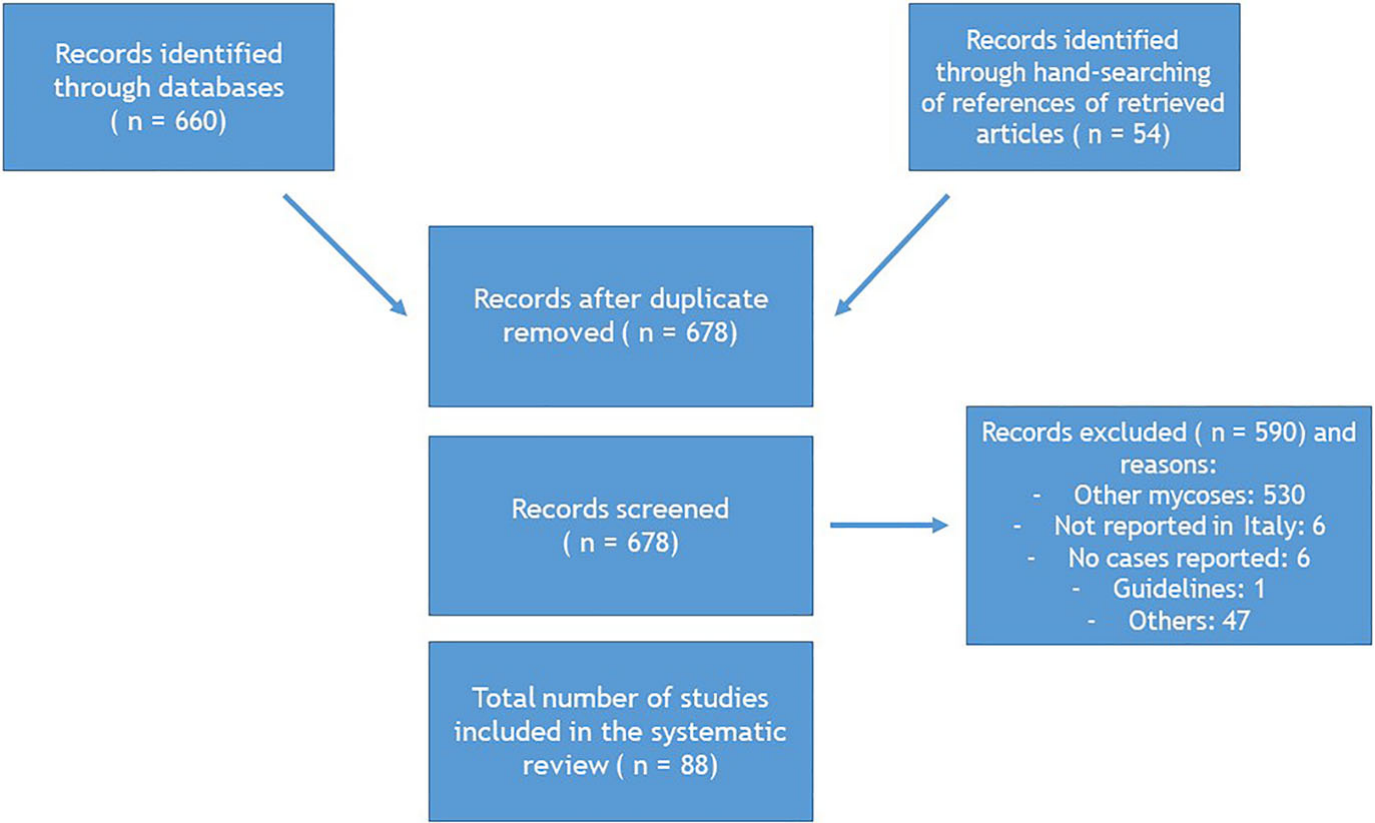
Literature selection procedure

The literature search identified 88 articles about cases of histoplasmosis, coccidioidomycosis, talaromycosis, blastomycosis, and paracoccidioidomycosis reported in Italy (Fig. 1).

### Talaromycosis

Talaromycosis, or penicilliosis, is a systemic fungal disease caused by *Talaromyces marneffei* (formerly *Penicillium marneffei*)*,* a dimorphic fungus classified in the group of the *Eurotiales* order and *Trichocomaceae* family [11]*.*

It is endemic in tropical and subtropical areas of South and Southeast Asia: in Northeastern India, Southern China, Hong Kong, Taiwan and Northern Thailand [11]*.* Talaromycosis especially affects immunocompromised patients, in particular those with HIV infection and a CD4 count < 100 cells/µL [12]. It has become the third most common HIV-related opportunistic infection in South and SouthEast Asia [12], where it is an AIDS-defining illness [11]. Travel-related talaromycosis is being increasingly reported also in non-endemic countries [11].

Human Infections occur through inhalation of *T. marneffei* conidia. The transmission is seasonal, coinciding with rainy seasons [11]. Bamboo rats are the main animal reservoir of *T. marneffei* [11]. There is no evidence of both animal-to-person or person-to-person transmission [12].

The incubation period of the disease is variable (from 1 to 3 weeks to years) [11]. Clinical manifestations appear only after hematogenous dissemination. The signs and symptoms are similar in adults and in children [13], but differ in patients with and without HIV. Patients without HIV are more likely to have bone and joint infections but are less likely to have fever, splenomegaly, skin lesions and positive fungal blood cultures [14]. Most patients present symptoms related to reticuloendothelial system involvement, including generalized lymphadenopathy, hepatomegaly, and splenomegaly. Talaromycosis can involve respiratory system (fever, dyspnea, and chest pain); gastrointestinal system (diarrhea); central nervous system (CNS) and cause mucosal or skin involvement (lesions appear as umbilicated papule, resembling molluscum contagiosum, nodules, or necrotic lesions, and they are usually located over the face and upper trunk) [15, 16].

The diagnosis is traditionally made through the identification of the fungus in clinical specimens by microscopy and culture [11]. Microscopically, *T. marneffei* appears as oval or round intracellular yeasts with cross-wall formations. Cultures of bone marrow, blood and biopsies of skin lesions have the highest sensitivity [12]. *Talaromyces marneffei* takes from 3 to 14 days to grow up in culture [11]. *Talaromyces marneffei* can be observed in histopathological sections with Grocott methenamine silver or periodic acid-Schiff stain. Infected tissues appear as granulomatous, suppurative reaction, and non-reactive necrosis [11]. No commercial serological tests are available. Antigens are available instead [3]. Polymerase chain reaction (PCR) based tests and next-generation sequencing (NGS) are useful tools for rapid diagnosis, even if hardly available. Galactomannan and β-D-glucan also can be helpful [11].

Disseminated disease is fatal if untreated. First choice treatment consists in liposomal amphotericin B (3–5 mg/kg per day) for 2 weeks, followed by oral itraconazole (200 mg/twice daily) for 10 weeks. If it is not possible to use amphotericin B, voriconazole or fluconazole (much less active) can be used. For immunocompromised patients itraconazole should be continued as secondary prophylaxis, until restoration of cellular immunity. For HIV patients with CD4 < 100 cells/µL living in endemic areas, primary prophylaxis with itraconazole 200 mg/day is indicated [3, 16].

Three cases of talaromycosis have been described in Italy (Table 1). All patients were immunocompromised (AIDS). Two patients were Italians who traveled to Thailand, the other one was a Chinese student living in Italy since 2016. All patients had disseminated infection, successfully treated with clinical recovery [17–19].

**Table 1:** Reported cases of talaromycosis, paracoccidioidomycosis, blastomycosis and coccidioidomycosis

Mycosis	Author, year	No patients	Nationality	Age, gender	Comorbidities	Immunocompromise	Country of infection acquisition
Talaromycosis	Antinori et al., 2006	1	Italian	36 y.o./M	Nº	Yes (AIDS)	Thailand
Talaromycosis	Viviani et al., 1993	1	Italian	33 y.o./M	Intravenous drug abuse; *Pneumocystis jiroveci *pneumonia (treated)	Yes (AIDS)	Thailand
Talaromycosis	Basile et al., 2022	1	Chinese	Late twenties /M	No	Yes (AIDS)	China
Paracoccidioidomycosis	Pecoraro et al., 1998	1	Venezuela	60 y.o./M	Smoker	No	Venezuela
Paracoccidioidomycosis	Scarpa et al., 1965	1	Italian	43 y.o./M	No	No	Venezuela
Paracoccidioidomycosis	Borgia et al., 2000	1	Venezuela	61 y.o./M	No	No	Venezuela
Paracoccidioidomycosis	Farris, 1955	1	Italian	66 y.o./M	No	No	Brazil
Paracoccidioidomycosis	Fulciniti et al., 1995	1	Italian	60 y.o./M	Smoker	No	Venezuela
Paracoccidioidomycosis	Benoldi et al., 1985	1	Italian	41 y.o./M	No	No	Venezuela
Paracoccidioidomycosis	Della Favera et al., 1914	1	Brazilian	13 y.o./M	Nd	Nd	Brazil
Paracoccidioidomycosis	Bertaccini et al., 1934	1	Italian	Nd	Nd	Nd	Brazil
Paracoccidioidomycosis	Molese et al., 1957	1	Italian	47 y.o/M	No	No	Venezuela
Paracoccidioidomycosis	Schirladi-Grimaldi, 1963	1	Italian	36 y.o. /M	No	No	Venezuela
Paracoccidioidomycosis	Velluti et al., 1979	1	Italian	52 y.o./M	No	No	Venezuela
Paracoccidioidomycosis	Lasagni et al., 1979	1	Italian	Nd	Nd	Nd	Venezuela
Paracoccidioidomycosis	Finzi et al., 1980	1	Italian	59 y.o. /M	No	No	Brazil
Paracoccidioidomycosis	Cuomo et al., 1985	1	Italian	37 y.o./M	Epilepsy	No	Venezuela
Paracoccidioidomycosis	Solaroli et al., 1998	1	Italian	49 y.o./M	No	No	Brazil
Blastomycosis	Codifava et al., 2012	1	Ghana	3 y.o. /M	No	No	Ghana
Blastomycosis	Ietto et al., 2021	1	Senegal	27 y.o. /M	Disseminated tuberculosis (treated)	No	Senegal
Blastomycosis	Rimondi et al., 1995	1	Italian	54 y.o. /M	No	No	None
Blastomycosis	Rivasi et al., 2000	1	Italian	78 y.o. /M	No	No	None
Blastomycosis	Rivasi et al., 2000	1	Italian	52 y. o. /M	No	No	None
Blastomycosis	Cavalot et al., 1992	1	Italian	57 y.o. /M	No	No	Central America
Blastomycosis	Sgobbi et al., 1978	1	Italian	31 y.o. /M	No	No	Canada
Blastomycosis	Wolf Chasen, 1951	1	Italian	25 y.o. /F	No	No	None
Blastomycosis	Wolf Chasen, 1951	1	Italian	19 y.o. /M	No	No	None
Blastomycosis	Florenzano-Zini, 1950	1	Italian	48 y.o. /F	Pulmonary tuberculosis	No	None
Coccidioidomycosis	Corpolongo et al., 2014	1	Italian	49 y.o. /M	No	No	Venezuela
Coccidioidomycosis	Tortorano et al., 2015	1	Italian	56 y.o. /F	No	No	Argentina
Coccidioidomycosis	D'Avino et al., 2012	1	Italian	48 y.o. /M	Disseminated cryptococcosis, CMV retinitis	Yes (AIDS)	United States
Coccidioidomycosis	Gobbi et al., 2012	1	Italian	28 y.o /M	Recurrent sinusitis	No	United States
Coccidioidomycosis	Scanarini et al., 1991	1	Italian	68 y.o /F	No	No	None
Coccidioidomycosis	Vilardo et al., 1964	1	Italian	43 y.o. /M	No	No	Venezuela
Coccidioidomycosis	Sotgiu and Corbelli, 1955	1	Italian	38 y.o. /M	No	No	United States
Coccidioidomycosis	Castellani, 1933	1	Italian	Nd	Nd	Nd	Nd
Coccidioidomycosis	Jacono-Boeri, 1932	1	Italian	Nd	Nd	Nd	Nd
Coccidioidomycosis	Jacono-Boeri, 1932	1	Italian	Nd	Nd	Nd	Nd

Mycosis	Clinical features	Species	Co-infection	Diagnosis	Treatment	Outcome	References
Talaromycosis	Disseminated (skin; bone marrow; blood)	*Talaromyces marneffei*	No	Positive culture of skin, bone marrow and blood; histology (skin)	Amphotericin B, then itraconazole	Recovered	[18]
Talaromycosis	Disseminated (skin; pnuemonia; blood)	*Talaromyces marneffei*	No	Positive culture of skin, sputum and blood; histology	Amphotericin B + flucitosine, then itraconazole	Recovered (died 1 year later for other opportunistic infections)	[17]
Talaromycosis	Disseminated (skin; brain; blood)	*Talaromyces marneffei*	No	Positive culture of blood; Histology; PCR	Amphotericin B, then isavuconazole	Recovered	[19]
Paracoccidioidomycosis	Osteomyelitis; subpleural nodular formations (one excavatum)	*Paracoccidioides brasiliensis*	No	Histology	Ketoconazole	Recovered	[57]
Paracoccidioidomycosis	Laterocervical lymphadenopathy; ulcerated lesions of the lip; dental avulsion; pulmonary: miliary dissemination, pneumonia with excavations, hilar and carenal lymphadenopathies and endotracheal vegetation	*Paracoccidioides brasiliensis*	No	Histology	Amphotericin B	Death	[63]
Paracoccidioidomycosis	Pneumonia; osteomyelitis	*Paracoccidioides brasiliensis*	No	Positive bone culture; histology	Itraconazole	Recovered	[59]
Paracoccidioidomycosis	Skin facial plaques; larynx; pneumonia	*Paracoccidioides brasiliensis*	No	Histology	Diathermocoagulation + sulfonamides	Persistency	[53]
Paracoccidioidomycosis	Osteomyelitis	*Paracoccidioides brasiliensis*	No	Histology	Itraconazole	Slow amelioration	[58]
Paracoccidioidomycosis	Ulcerated skin plaque; pneumonia	*Paracoccidioides brasiliensis*	No	Histology; serology	Ketoconazole + sulfamethoxypyridazine	Recovered	[62]
Paracoccidioidomycosis	Nd	*Paracoccidioides brasiliensis*	Nd	Nd	Nd	Nd	[52]
Paracoccidioidomycosis	Nd	*Paracoccidioides brasiliensis*	Nd	Nd	Nd	Nd	[56]
Paracoccidioidomycosis	Pneumonia; oral mucosa and lymph nodes involvement; hepatosplenomegaly	*Paracoccidioides brasiliensis*	No	Histology	Nystatin	Persistency	[61]
Paracoccidioidomycosis	Generalized lymphadenopathy; Gastro-intestinal	*Paracoccidioides brasiliensis*	No	Positive culture of lymph nodes and feces; histology	Nystatin, then amphotericin B	Recovered	[64]
Paracoccidioidomycosis	Pneumonia	*Paracoccidioides brasiliensis*	No	Nd	Amphotericin B and miconazole	Recovered	[60]
Paracoccidioidomycosis	Nd	*Paracoccidioides brasiliensis*	Nd	Nd	Nd	Nd	[60]
Paracoccidioidomycosis	Pneumonia; skin involvement	*Paracoccidioides brasiliensis*	No	Histology	Miconazole	Recovered	[55]
Paracoccidioidomycosis	Pneumonia; skin involvement	*Paracoccidioides brasiliensis*	No	Histology; serology	Ketoconazole	Recovered	[60]
Paracoccidioidomycosis	Pneumonia; brain and skin involvement	*Paracoccidioides brasiliensis*	No	Nd	Itraconazole	Recovered	[54]
Blastomycosis	Osteolytic lesion of the distal femur and muscle abscess	*Blastomyces dermatitidis*	No	Histology	Surgery + Amphotericin B, then itraconazole	Recovered	[25]
Blastomycosis	Fluid collections in psoas muscles with bone lytic lesions of the pelvis	*Blastomyces dermatitidis*	No	PCR; sequencing of the 18 s region	Itraconazole	Recovered	[34]
Blastomycosis	Adrenal insufficiency	*Blastomyces dermatitidis*	No	Positive culture of adrenal glands biopsy; histology	Fluconazole	Recovered	[40]
Blastomycosis	Nodule on the right knee	*Blastomyces dermatitidis*	No	Histology	Nd	Nd	[38]
Blastomycosis	Nodule on the right knee	*Blastomyces dermatitidis*	No	Histology	Nd	Nd	[38]
Blastomycosis	Infiltrative lesions of the lip, tongue, soft palate and epiglottis; larynx	*Blastomyces dermatitidis*	No	Histology	Amphotericin B, then itraconazole	Recovered	[35]
Blastomycosis	Pneumonia; spondylodiscitis	*Blastomyces dermatitidis*	No	Histology	Amphotericin B	Recovered	[36]
Blastomycosis	Gum nodule; odontopathy	*Blastomyces dermatitidis*	No	Microscopic detection	Potassium iodide + surgery	Recovered	[39]
Blastomycosis	Gum nodule	*Blastomyces dermatitidis*	Streptococcal skin infection	Microscopic detection	Potassium iodide + surgery + penicillin	Recovered	[39]
Blastomycosis	Pneumonia	*Blastomyces dermatitidis*	No	Microscopic detection	Nd	Nd	[37]
Coccidioidomycosis	Pneumonia	*Coccidioides immitis*	No	Serology	Fluconazole	Recovered	[75]
Coccidioidomycosis	Persistent erythematous papular plaque	*Coccidioides posadasii*	No	Microscopic detection; culture; histology	Itraconazole	Lost to follow up	[74]
Coccidioidomycosis	Lymphadenopathy	*Coccidioides immitis*	No	Histology	Fluconazole	Recovered	[71]
Coccidioidomycosis	Pneumonia	*Coccidioides immitis*	No	Serology; microscopic detection; positive culture of Bronchoalveolar lavage	Itraconazole	Recovered	[72]
Coccidioidomycosis	Pituitary granuloma	*Coccidioides immitis*	No	Histology	Surgery + ketoconazole	Recovered	[76]
Coccidioidomycosis	Pneumonia; oral involvement + Lymphadenopathy	*Coccidioides immitis*	No	Histology	Amphotericin B	Amelioration	[77]
Coccidioidomycosis	Pneumonia	*Coccidioides immitis*	No	Microscopic detection; culture	Nd	Amelioration	[73]
Coccidioidomycosis	Nd	Nd	Nd	Nd	Nd	Nd	[73]
Coccidioidomycosis	Nd	Nd	Nd	Nd	Nd	Nd	[73]
Coccidioidomycosis	Nd	Nd	Nd	Nd	Nd	Nd	[73]

*CMV* Cytomegalovirus

### Blastomycosis

Blastomycosis is a systemic fungal disease caused by *Blastomyces dermatitidis,* a dimorphic fungus classified in the group of the *Onygenales* order and *Ajellomycetaceae* family [20]. *Blastomyces gilchristii* is another species identified in 2013, which causes similar disease in humans [21]. Recently another distinct species was identified in Canada: *Blastomyces helicus* [22].

Blastomycosis is endemic in the United States of America (USA) and Canada, particularly in the Mississippi and Ohio River valleys, in the Mid-western states and Canadian provinces that border the Great Lakes, and areas adjacent to the Saint Lawrence Seaway. *B. dermatitidis* is also present in Africa and India [23, 24]. No epidemiological data are available for Europe, where the disease has been reported only in travelers [25].

*Blastomyces* species are found in a specific ecological niche characterized by wet earth with animal droppings (especially dogs) and decaying vegetation [26, 27]. Infection in people occurs through inhalation of the conidia. Sometimes, the infection could be transmitted through direct skin inoculation (for trauma or insect bites). *B. dermatitidis* is usually not transmitted from person to person and not even from animal to person [26].

Approximately 50% of people exposed to blastomycosis develop symptoms, whereas the remaining 50% have asymptomatic or subclinical disease. Incubation period of blastomycosis ranges from 3 weeks to 3 months [28]. Pulmonary blastomycosis can present with acute pneumonia which mimic bacterial pneumonia or chronic pneumonia like tuberculosis and lung cancer [26]. Extrapulmonary dissemination (via hematogenous spread or direct inoculation) affects skin, bones, and male genitourinary system, in decreasing order of frequency. Skin lesions are frequently misdiagnosed as pyoderma gangrenosum or basal/squamous cell carcinoma. CNS involvement is uncommon in immunocompetent hosts; it may occur in HIV patients, either as meningitis or cranial abscesses [29].

The gold standard for diagnosis is the culture of clinical specimens. Growth on Sabouraud dextrose agar is very slow and could take up to four weeks. For this reason histopathological identification is very important. Yeast appears as 15 µm cells with thick, double-refractile walls and a single broad-based bud. Gomori methenamine silver or periodic–acid Schiff staining are usually used for tissue samples, calcofluor white or Papanicolaou stains are used for respiratory samples. Antigen testing (not commercially available as a kit) of the galactomannan component can be performed on urine, serum, bronchoalveolar lavage, and cerebrospinal fluid (CSF). Antigenuria has a sensitivity of 75–93% and specificity of 80% in patients with proved blastomycosis. Cross-reactivity is described for histoplasmosis, paracoccidioidomycosis, and talaromycosis [30]. Serum β-d-glucan is unreliable for diagnosis of blastomycosis because the yeast cell wall contains very little of this carbohydrate [31]. Serological tests (complement fixation or immunodiffusion) are available, but they have poor sensitivity and specificity. PCR based tests are useful (even if they are still not validated) [30].

Itraconazole is the first choice for treatment in all forms (600 mg/day for 3 days, then 200–400 mg/day for 6/12 months), except in severe or life-threatening diseases. In these cases, liposomal amphotericin B (3–5 mg/kg/day) is indicated until clinical improvement, followed by itraconazole 200 mg twice daily for 6–12 months [32]. CNS blastomycosis should be treated for at least 12 months and until CNS abnormalities resolution (liposomal amphotericin B 5 mg/kg/day for 4–6 weeks, followed by an oral azole) [33].

Ten cases of blastomycosis have been described in Italy (Table 1). All patients were immunocompetent. In all cases, the species identified was *B. dermatitidis*. Nevertheless as all but one of the cases were diagnosed before 2013, one cannot disregard misidentifications with the recently described species *B. gilchristii* and *B. helicus*. Only four subjects have a travel history in these countries, respectively: Ghana [25], Senegal [34], Central America [35] and Canada [36]. Only two patients developed pneumonia [36, 37], the other eight presented with extrapulmonary manifestations: bone, joints and soft tissues (n = 5) [25, 34, 36, 38], oral cavity (n = 3) [35, 39] and adrenal glands (n = 1) [40]. No deaths are reported. Seven patients recovered (for three patients this information is lacking).

### Paracoccidioidomycosis

Paracoccidioidomycosis is a systemic fungal disease caused by *Paracoccidioides* spp, a dimorphic fungus classified in the group of the *Onygenales* order and *Ajellomycetaceae* family. Two species, *P. brasiliensis *sensu stricto and *P. lutzii,* cause paracoccidioidomycosis. Recently other species have been described: *P. americana*, *P. restrepiensis* and *P. venezuelensis* [41–43].

Paracoccidioidomycosis is endemic in Latin America, especially in Brazil, Colombia and Venezuela. Argentina (North), Ecuador (Cuenca River valley), and Paraguay (Oriental side) are areas of moderate to high endemicity. Southern Mexico, from the Gulf of Mexico to the Pacific Coast, and Central American countries are territories of low endemicity. Few data are available for Bolivia, Peru, and Uruguay, but autochthonous cases have been reported [44]. Cases of paracoccidioidomycosis have been reported in Europe, United States, Canada, Japan, Africa, and the Middle East. All of them affected patients who visited or lived in South America [9, 44].

Paracoccidioidomycosis is a noncontagious disease, transmitted through conidia inhalation from soil and humid vegetation. Men and armadillos are the main accidental hosts [44].

*Paracoccidioides* species usually causes a subclinical primary pneumonia [45]. Only 1–2% of infected individuals develop clinical manifestations during their lives [46]. We can distinguish two main clinical forms of paracoccidioidomycosis: the acute or subacute form (juvenile type) and the chronic form (adult type) [45]. In endemic area, the acute/subacute form occurs in children, youths, and in adults under 30 years of age [45], but travelers can show similar clinical presentation. In this case the disease develops a few weeks or months after exposure to *Paracoccidioides* spp [44]. Dissemination is possible and typically causes fever, lymphadenopathy, and hepatosplenomegaly. Intestinal, cutaneous and neurological involvement is also possible, while lung involvement is rare [45, 46]. The chronic form affects mostly adult males and it usually consists of a reactivation of pulmonary latent foci formed during the primary infection [45], presenting with cough, dyspnea, and sputum expectoration [46]. The chronic form occurs many years after exposure to *Paracoccidioides* spp [44]. Dissemination is possible and typically affects lymph nodes, skin, adrenal glands, CNS, and oral mucosa [45].

The gold standard for diagnosis is the identification of the fungus in clinical specimens [44]. It appears as spherical yeast, with multiple budding yeasts surrounding a mother cell and birefringent and greenish walls [47]. *P. brasiliensis* cultures usually take weeks to grow [48]*.* Histological examination through Grocott–Gomori staining shows granuloma with giant multinucleated cells and polymorphonuclear cell infiltrates [47]. Several immunoassays are available[47]. The most used is double agar gel immunodiffusion because of its cost-effectiveness, sensitivity (> 80%) and specificity (> 90%) [44]. Serum β-d-glucan has good sensitivity but it seems to be not useful for predicting clinical response to antifungal therapy [49]. *Paracoccidioides* spp can be also detected by PCR based test [47].

The first choice for treatment is itraconazole (200 mg/day administered for 6–9 months in mild disease and for 12–18 months in moderate disease) [50]. Trimethoprim-sulfamethoxazole requires a longer duration of therapy (at least 24 months), with lower cure rates, and higher relapse rates when compared to itraconazole [51]. Liposomal amphotericin B is indicated for CNS disease, severe/disseminated forms and in immunocompromised patients (3–5 mg/kg/day, followed by oral azole or trimethoprim-sulfamethoxazole) [50]. For immunocompromised patients itraconazole should be continued until restoration of cellular immunity (secondary prophylaxis). Primary prophylaxis with trimethoprim-sulfamethoxazole is effective also for paracoccidioidomycosis [50].

Fifteen cases of paracoccidioidomycosis have been described in Italy since 1914 (Table 1) [52]. All patients have traveled or lived in South America: 10 patients in Venezuela and the other 5 in Brazil [52–56]. Lung involvement was described in 10 patients. The other presented extrapulmonary manifestations related to bone (3) [57–59], skin and mucosa (6) [53, 54, 60–63] and lymph nodes (3) [61, 63, 64]. All patients were immunocompetent. Death occurs only in one case [63], an italian farmer who worked for four years in Venezuela and developed after five years chronic pulmonary paracoccidioidomycosis, disseminated to lymph nodes, skin and oral mucosa.

### Coccidioidomycosis

Coccidioidomycosis, also known as ‘‘Valley fever’’[65], is a systemic fungal infection caused by some dimorphic fungi belonging to the genus *Coccidioides* which is classified in the *Onygenales* order and the *Onygenaceae* family [20]. Only two species, *Coccidioides immitis* and *Coccidioides posadasii*, have been differentiated [65].

*Coccidioides* species are endemic in the deserts of the North and in some areas of Central and South America [66]. In the United States, California and Arizona are the states with the most of the cases [67]*.*

The transmission of coccidioidomycosis is seasonal. The highest incidence occurs in the fall. Coccidiomycosis is not transmitted from person to person but only through conidia inhalation from soil [66].

Primary pulmonary coccidioidomycosis is the typical clinical presentation of the disease. It could be asymptomatic, mild to moderate (often resolved without treatment) or severe. Some individuals develop pulmonary complications as pleural effusions, cavitations, fibrocavitations, and empyema. Extrapulmonary dissemination occurs in a small percentage of patients (HIV/AIDS patients have higher risk). Dissemination is described typically in these sites: bone, skin and soft tissues, CNS, and lymph nodes. Coccidioidal meningitis is fatal, when untreated [66].

**Fig. 2:**
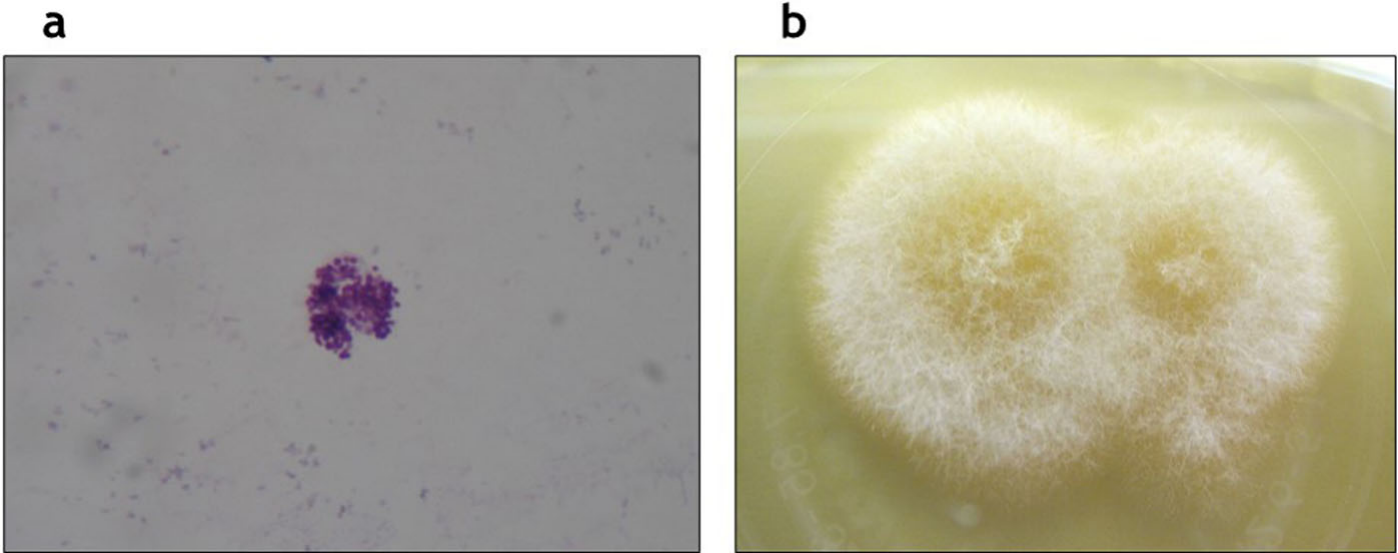
*Coccidioides immitis*. **A** Coccidial spherules through direct microscopy. **B** Colonies grown on Sabouraud dextrose agar after three days of incubation. [Courtesy of Chiara Savio]

Coccidioidomycosis can be diagnosed by direct microscopic examination and culture of clinical specimens (Fig. 2a) [66]. Colonies of *Coccidioides* spp develop readily, usually within 3–5 days, on Sabouraud dextrose agar (Fig. 2b) [3, 68]. Through direct microscopy coccidial spherules are observed. Mature spherules are thick-walled (80 μm diameter), with endospores (2–4 μm diameter) inside [68]. Histological examination shows granulomatous inflammation. PCR based tests are also available [66]. Pulmonary coccidioidomycosis could be diagnosed by a serological test, with the immunodiffusion for the detection of precipitating specific antibodies (IgM are produced 1–3 weeks after symptoms onset, followed by IgG 4–8 weeks later) [3]. Skin tests are also feasible. Their positivity indicates past or present infection. Skin test conversion indicates infection in the intervening time [66]. Coccidioidal antigen with an enzyme immunoassay test is available (not commercially available as a kit) [3]. β-d-glucan can be useful, even if it has low sensitivity [69].

Appropriate management of coccidioidomycosis requires treatment with fluconazole (400 mg/day orally) or itraconazole (200 mg 2 times daily) for patients with symptomatic chronic cavitary pneumonia, with soft tissue and bone involvement, immunocompromise and meningitis. Surgery may be considered in patients not responsive to treatment. For CNS disease the treatment is long-life (higher dosages are required). For immunocompromised patients, itraconazole should be continued until restoration of cellular immunity (secondary prophylaxis). For HIV patients in endemic areas primary prophylaxis is not indicated, but serological screening is recommended. Severe disease should be treated with liposomal amphotericin B (3–5 mg/kg/day) [70].

Ten cases of coccidioidomycosis have been described in Italy (Table 1). In particular, three cases were imported from the USA [71–73], while others were from South America (Argentina and Venezuela) [74, 75]. In four cases no travel history was reported [76]. All patients were Italian citizens. Only one case was attributed to *C. posadasii* [74], while in the other cases *C. immitis* was detected as an etiologic agent. Only one patient was immunocompromised (AIDS) [71]. No deaths were reported. In 1955 Sotgiu and Corbelli described a case of pneumonia in an Italian man who worked in the port of Genoa, who stayed in close contact with grain from North America. In this paper the authors referred to this case as the 4th described in Italy, after Castellani, Jacono and Boeri in 1933 and 1932 [73]. In 1991 Scanarini et al. reported a rare case of primary intrasellar localization of coccidioidomycosis [76]. Gobbi et al. in 2012 reported the case of a 28-years-old Italian man living in Tucson, Arizona for study purposes. He traveled in California and Nevada for visiting Sonora Desert and developed pneumonia, once coming back to Italy [72]. Tortorano et al. (2015) reported a case of a 56-years-old man with persistent erythematous papular plaque without other symptoms [74]. D'Avino et al. in 2012 reported a rare case of AIDS patient with cervical-node coccidiomycosis [71]. Four patients fully recovered [71, 72, 75, 76], two showed clinical improvement [73, 77]. For the other patients no follow-up data are available.

### Histoplasmosis

Histoplasmosis is a systemic fungal disease caused by *Histoplasma capsulatum,* a dimorphic fungus classified in the group of the *Onygenales* order and *Ajellomycetaceae* family.

Three different varieties have been historically recognised, each with a typical geographic distribution: *H. capsulatum* var. *capsulatum* (New World, mainly in the Ohio and Mississippi River Valleys in USA, and Latin America), responsible for classic human histoplasmosis, *H. capsulatum* var. *duboisii* (Central and West Africa), responsible for African human histoplasmosis, *H. capsulatum* var. *farciminosum* (Old World, mainly in Asia) responsible for histoplasmosis in equines [78, 79]. Recent studies demonstrated that *Histoplasma* genus shows high diversity and four geographical clusters have been proposed for american isolates (*H. capsulatum *sensu stricto, *H. ohiense*, *H. suramericanum*, and *H. mississippiense*), but there is no consensus today on these new species [79, 80]. Some histoplasmin skin reports and case reports showed that histoplasmosis is distributed more extensively than historically thought and probably has ecological niches also in Italy and Europe [81, 82].

Histoplasmosis is transmitted through conidia inhalation from soil containing bird or bat guano [83]. It is usually not transmitted from person to person, even if transmission through solid organ transplant is reported [84].

After inhalation, about 90% of individuals exposed remain asymptomatic or develop self-limited symptoms. Pulmonary histoplasmosis can be acute, subacute, and chronic with cavitations or nodules. The incubation period is short for the acute disease (2 weeks). Dissemination is described typically in HIV/AIDS patients in: skin and soft tissues, CNS, bone marrow and lymph nodes. Physical examination usually reveals lymphadenopathy, hepatomegaly, and splenomegaly [83]. Histoplasmosis is an AIDS-defining illness [85]. The progressive disseminated form has high mortality. Skin involvement implicates diffuse maculopapular eruption and ulceration in advanced lesions [84].

**Fig. 3:**
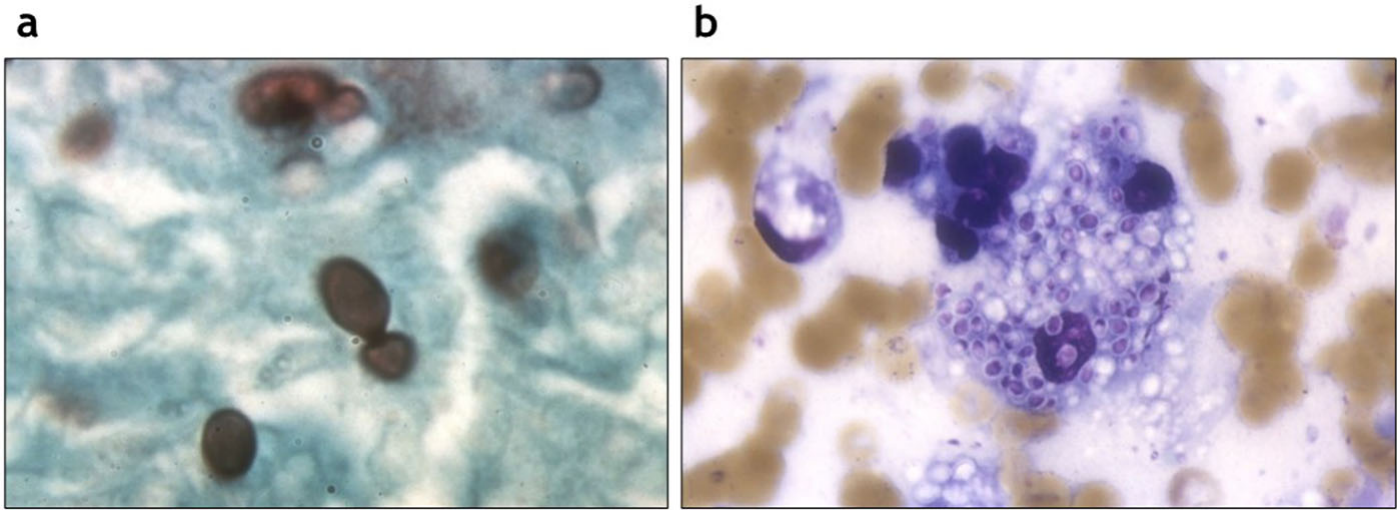
*Histoplasma capsulatum*. **A** Yeast cells with Gomori methenamine silver stain. **B** Blastoconidia inside a macrophage (Giemsa stain). [Courtesy of Stefano Andreoni, Claudio Farina, Gianluigi Lombardi]

The culture of clinical specimens is routinely used for the diagnosis. Growth in Sabouraud dextrose agar culture usually takes up from 2 to 8 weeks [86]. Cultures of bone marrow, blood and respiratory samples are usefull in disseminated infection and in chronic cavitary pulmonary disease, respectively. CSF culture is often negative [84]. Histopathological evidence of *H. capsulatum* is one of the diagnostic criteria of proven histoplasmosis [87]. The yeast cells are ovoidal (size 2–4 μm), usually intracellular and showing narrow-based budding with Gomori methenamine silver (Fig. 3a) or periodic–acid Schiff stains. Histoplasmosis typically presents granulomatous inflammation (Fig. 3b), which may be caseating or non caseating [84]. Serological tests are available and show the highest sensitivity in subacute and chronic histoplasmosis [86]. Histoplasmosis antigen testing is mostly done on urine, but it is available (even if not validated) also on serum, bronchoalveolar lavage, and CSF. It’s useful also for monitoring the effectiveness of the therapy. The sensitivity is high in HIV/AIDS patients and in the disseminated disease (antigen detected in 95% of cases) [84]. For CNS histoplasmosis diagnosis the best combination is made by serology and CSF antigen tests [88]. Urine test for histoplasmosis antigen is indicated in patients with CD4 count < 100 cells/µL in areas where histoplasmosis is endemic, and it must be repeated every year [89]. Histoplasmosis antigen is not available worldwide (also in Italy). Cross-reactions occur with other endemic mycoses both for serologic tests and antigens [84, 86]. Serum β-d-glucan is not reliable for histoplasmosis diagnosis [31]. PCR based tests are useful (even if they are still not validated) [84].

Chronic pulmonary and disseminated histoplasmosis are fatal if untreated [84]. International guidelines are available [90]. The first choice for treatment is itraconazole (600 mg/day for 3 days, then 200–400 mg/day for 12 months) for mild/moderate acute pulmonary histoplasmosis and for chronic cavitary pulmonary. For severe pulmonary disease intravenous steroids are recommended [90]. Liposomal amphotericin B is indicated in CNS disease, severe/disseminated forms and in immunocompromised patients (3–5 mg/kg/day) for 2 weeks, followed by itraconazole for at least 12 months. For immunocompromised patients itraconazole should be continued until restoration of cellular immunity and demonstration of clinical cure (secondary prophylaxis) [91]. Primary prophylaxis with itraconazole 200 mg/day is indicated for HIV patients with CD4 < 150 cells/μL living in endemic areas [84].

Systematic reviews have been conducted about cases of histoplasmosis in Italy [4, 6–8]. According to our systematic research, 105 cases of histoplasmosis have been described in Italy (Table 2). Death occurs in 20 patients (19%). For 28 patients no history of travels outside Italy was described. For all the other subjects a country of infection's acquisition was signaled. Most cases have been acquired in Ecuador (n = 20) [92–95] and in general in central and south America [96, 97]. Immunocompromised patients were 49:42 with HIV/AIDS, two with leukemia [98], one with breast cancer [99], one has Crohn disease [94], one sarcoidosis [94], one rheumatoid arthritis [100] and only one was a lung transplant recipient [101]. Among them, 14 deaths were reported (28%). Almost half of the patients (n = 48) presented disseminated histoplasmosis. Most of them had AIDS as a major risk factor. Unusual clinical presentations have also been reported: one case of endophthalmitis occurred in an Italian 64 years old man with diabetes who lived in Brazil for several years [102], and one of adrenal incidentaloma [103] in an Italian 74-year-old man who worked in Pakistan as a well-driller for 2 years. Two different clusters have been investigated. In 1997 Nasta et al. described the first one. Four Italian spelunkers, returning from Perù, presented mild acute pulmonary disease with hepatosplenomegaly with complete resolution after ketoconazole treatment [104]. The second consists of 17 members of a naturalistic expedition to Ecuador. All subjects were immunocompetent, only one presented disseminated histoplasmosis, while the other suffered from mild acute pulmonary disease. All of them recovered. Only seven patients required antifungal therapy [92].

**Table 2:** Reported cases of histoplasmosis

Mycosis	Author, year	No patients	Nationality	Age, gender	Comorbidities	Immunocompromise	Country of infection acquisition
Histoplasmosis	Sotgiu and Corbelli, 1955	1	Italian	60 y.o. /M	Nd	Nd	Nd
Histoplasmosis	Sotgiu and Corbelli, 1955	1	Italian	31 y.o. /M	Nd	Nd	Nd
Histoplasmosis	Corbelli et al., 1957	1	Italian	68 y.o. /M	Nd	Nd	Nd
Histoplasmosis	Corbelli et al., 1957	1	Italian	32 y.o. /M	Nd	Nd	Nd
Histoplasmosis	Zavoli, 1957	1	Italian	39 y.o. /M	Recurrent tonsillitis, angina	No	India, East Africa
Histoplasmosis	Allegri and Bottiglioni, 1958	1	Italian	42 y.o. /F	Nd	Nd	Nd
Histoplasmosis	Costa et al., 1959	1	Italian	24 y.o. /M	Nd	Nd	Nd
Histoplasmosis	Salfelder et al., 1963	1	Italian	25 y.o. /F	Nd	Nd	Nd
Histoplasmosis	Papa et al., 1965	1	Italian	66 y.o. /M	No	No	None
Histoplasmosis	Mesolella et al., 1966	1	Italian	71 y.o. /M	No	No	None
Histoplasmosis	Altucci et al., 1968	1	Italian	54 y.o. /F	Nd	Yes (leukemia)	None
Histoplasmosis	Altucci et al., 1968	1	Italian	72 y.o. /F	Nd	Yes (leukemia)	None
Histoplasmosis	Pellegrino et al., 1977	1	Nd	Nd	Nd	No	None
Histoplasmosis	Zanini et al., 1987	1	Italian	60 y.o. /M	Malaria (treated), edentulous	No	Africa (Nigeria, Guinea, Camerun)
Histoplasmosis	Masini et al., 1988	1	Nd	Nd	Nd	Yes (AIDS)	Nd
Histoplasmosis	Vaj et al., 1989	1	Italian	31 y.o. /M	No	No	Mexico
Histoplasmosis	Visonà et al., 1991	1	Italian	29 y.o. /M	No	No	Ecuador
Histoplasmosis	Tinelli et al., 1992	1	Italian	37 y.o. /M	No	No	Africa (Nigeria, Sudan, Zaire)
Histoplasmosis	Biglino et al., 1992	1	Italian	41 y.o. /F	No	No	None
Histoplasmosis	Gori et al., 1993	1	Italian	48 y.o. /M	No	Yes (AIDS)	America
Histoplasmosis	Confalonieri et al., 1994	1	Italian	50 y.o. /M	Smoker, mycosis fungoides (treated with chemotherapy)	No	None
Histoplasmosis	Confalonieri et al., 1994	1	Italian	54 y.o. /M	Smoker	No	None
Histoplasmosis	Manfredi et al., 1994	1	Italian	29 y.o. /M	IDU	Yes (AIDS)	Mexico
Histoplasmosis	Gargiulo et al., 1995	1	Ivorian	36 y.o. /M	Nd	Yes (AIDS)	Ivory Coast
Histoplasmosis	Conte et al., 1996	1	Brazilian	28 y.o. /M	No	Yes (AIDS)	America
Histoplasmosis	Vullo et al., 1997	1	Italian	36 y.o. /M	IDU	Yes (AIDS)	United States
Histoplasmosis	Antinori et al., 1997	1	Italian	35 y.o. /M	Spinocellular carcinoma (treated)	Yes (AIDS)	None
Histoplasmosis	Nasta et al., 1997	1	Italian	Late twenties /M	No	No	Perù
Histoplasmosis	Nasta et al., 1997	1	Italian	Late twenties /M	No	No	Perù
Histoplasmosis	Nasta et al., 1997	1	Italian	Late twenties /M	No	No	Perù
Histoplasmosis	Nasta et al., 1997	1	Italian	Late twenties /M	No	No	Perù
Histoplasmosis	Angius et al., 1998	1	Argentinian	53 y.o. /M	Kaposi Sarcoma	Yes (AIDS)	Argentina
Histoplasmosis	Pometta et al., 1999	1	Italian	41 y.o. /M	No	No	San Salvador
Histoplasmosis	Faggi et al., 2000	1	Brazilian	24 y.o. /M	No	Yes (AIDS)	Brazil
Histoplasmosis	D'Antuono et al., 2000	1	Nd	Nd	Nd	Yes (AIDS)	Nd
Histoplasmosis	Antinori et al., 2000	1	Brazilian	35 y.o. /M	No	Yes (AIDS)	Brazil
Histoplasmosis	Antinori et al., 2000	1	Italian	29 y.o. /F	IDU, Herpes Zoster (treated)	Yes (AIDS)	None
Histoplasmosis	Farina et al., 2000	1	Italian	43 y.o. /M	IDU, HCV chronic infection	Yes (AIDS)	None
Histoplasmosis	Farina et al., 2000	1	Italian	22 y.o. /M	No	No	Guatemala; Honduras
Histoplasmosis	Farina et al., 2000	1	Venezuelan	50 y.o. /F	Lue (treated)	Yes (AIDS)	Venezuela
Histoplasmosis	Farina et al., 2000	1	Italian	29 y.o. /M	No	No	Perù
Histoplasmosis	Farina et al., 2000	1	Italian	32 y.o. /M	IDU	Yes (AIDS)	Nepal; India; Marocco
Histoplasmosis	Farina et al., 2000	1	Italian	45 y.o. /F	No	No	Dominican Republic
Histoplasmosis	Farina et al., 2000	1	Italian	53 y.o. /F	No	No	Dominican Republic
Histoplasmosis	Romano et al., 2000	1	Italian	80 y.o. /M	Hypertension	Yes (rheumatoid arthritis)	None
Histoplasmosis	Lio et al., 2000	1	Italian	74 y.o. /M	Alcohol abuser	No	Pakistan
Histoplasmosis	Mignogna et al., 2001	1	Tanzanian	44 y.o. /M	No	No	Tanzania; United States
Histoplasmosis	Rizzi et al., 2001	1	Nd	Nd	IDU	Yes (AIDS)	None
Histoplasmosis	Rizzi et al., 2001	1	Nd	Nd	IDU	Yes (AIDS)	None
Histoplasmosis	Rivasi et al., 2001	1	Ghana	36 y.o. /M	Malaria, Herpes Zoster and Candida esophagitis (treated)	Yes (AIDS)	Ghana
Histoplasmosis	Calza et al., 2003	1	Italian	43 y.o. /M	IDU	No	None
Histoplasmosis	Lo Cascio et al., 2003	1	Nigeria	40 y.o. /F	Nephrotic syndrome	Yes (AIDS)	Nigeria
Histoplasmosis	Faggian et al., 2004	1	Nigeria	40 y.o. /F	Nephrotic syndrome	Yes (AIDS)	Nigeria
Histoplasmosis	Faggian et al., 2004	1	Colombian	29 y.o. /M	Pulmonary tuberculosis	Yes (AIDS)	Colombia
Histoplasmosis	Garavelli et al., 2005	1	Colombian	28 y.o. /M	Kaposi Sarcoma	Yes (AIDS)	Colombia
Histoplasmosis	Farina et al., 2005	1	Italian	45 y.o. /M	No	No	Nicaragua
Histoplasmosis	Farina et al., 2005	1	Ivorian	37 y.o./F	No	Yes (AIDS)	Ivory Coast
Histoplasmosis	Farina et al., 2005	1	Ivorian	48 y.o. /M	No	Yes (AIDS)	Ivory Coast
Histoplasmosis	Farina et al., 2005	1	Italian	41 y.o. /F	No	Yes (AIDS)	None
Histoplasmosis	Antinori et al., 2006	1	Ivorian	30 y.o. /M	Herpes Zoster (treated)	Yes (AIDS)	Ivory Coast
Histoplasmosis	Antinori et al., 2006	1	Brazilian	29 y.o. /F	No	Yes (AIDS)	Brazil
Histoplasmosis	Antinori et al., 2006	1	Brazilian	29 y.o. /M	PJP (treated)	Yes (AIDS)	Brazil
Histoplasmosis	Antinori et al., 2006	1	Italian	42 y.o. /M	IDU	Yes (AIDS)	South America
Histoplasmosis	Galetta et al., 2007	1	Italian	64 y.o. /F	Malaria (treated)	Yes (Breast cancer)	Costa Rica
Histoplasmosis	Bartoloni et al., 2011	1	Ecuadorian	35 y.o. /M	No	Yes (AIDS)	Ecuador
Histoplasmosis	Inojosa et al., 2011	1	Ghana	30 y.o. /M	Hypertension	Yes (AIDS)	Ghana
Histoplasmosis	Inojosa et al., 2011	1	Liberia	32 y.o. /M	No	Yes (AIDS)	Liberia
Histoplasmosis	Inojosa et al., 2011	1	Senegal	47 y.o. /M	PJP (treated), HBV chronic infection	Yes (AIDS)	Senegal
Histoplasmosis	Inojosa et al., 2011	1	Ivorian	40 y.o. /F	No	Yes (AIDS)	Ivory Coast
Histoplasmosis	Fortuna et al., 2011	1	Italian	67 y.o. /M	No	No	None
Histoplasmosis	Scarlata et al., 2011	1	Ghana	36 y.o. /M	No	Yes (AIDS)	Ghana
Histoplasmosis	Grancini et al., 2013	1	Italian	64 y.o. /M	Diabetes with retinopathy, chronic renal failure, cardiovascular disease	No	Brazil
Histoplasmosis	Ardizzoni et al., 2013	1	Italian	30 y.o. /M	No	No	Brazil
Histoplasmosis	Righi et al., 2014	1	Italian	63 y.o. /M	Sarcoidosis	Yes (Lung transplant)	None
Histoplasmosis	Amadori et al., 2015	1	Brazilian	24 y.o. /M	No	Yes (AIDS)	Brazil
Histoplasmosis	Amadori et al., 2015	1	Thailand	39 y.o. /F	HBV and HCV hepatitis	Yes (AIDS)	Thailand
Histoplasmosis	Delfino et al., 2015	1	Ecuadorian	32 y.o. /M	Pulmonary tuberculosis (incompletely treated)	Yes (AIDS)	Ecuador
Histoplasmosis	Bonsignore et al., 2017	1	Italian	43 y.o. /F	Splenectomy	No	None
Histoplasmosis	Zanotti et al., 2018	1	Ivorian	19 y.o. /F	No	Yes (AIDS)	Ivory Coast
Histoplasmosis	Papalini et al., 2019	1	Cuban	33 y.o. /F	No	Yes (AIDS)	Cuba
Histoplasmosis	Staffolani et al., 2020	17	Nd	11/17 male; 38.5 mean age (years)	Nd	No	Ecuador
Histoplasmosis	Staffolani et al., 2020	1	Nd	Nd	No	No	Bolivia
Histoplasmosis	Staffolani et al., 2020	1	Nd	Nd	No	No	Mexico
Histoplasmosis	Staffolani et al., 2020	1	Nd	Nd	No	No	Mexico
Histoplasmosis	Staffolani et al., 2020	1	Nd	Nd	No	No	Cuba
Histoplasmosis	Staffolani et al., 2020	1	Nd	Nd	Nd	Yes (Crohn Disease)	Panama
Histoplasmosis	Staffolani et al., 2020	1	Nd	Nd	Nd	Yes (Sarcoidosis)	South America
Histoplasmosis	Asperges et al., 2021	1	Colombian	42 y.o. /F	No	Yes (AIDS)	Colombia
Histoplasmosis	Antinori et al., 2021	1	Brazilian	27 y.o. /M	Disseminated tuberculosis (treated)	Yes (AIDS)	Brazil

Mycosis	Clinical features	Species	Co-infection	Diagnosis	Treatment	Outcome	References
Histoplasmosis	Primary pulmonary	*Histoplasma capsulatum*	Nd	Positive culture of blood; histology	Nd	Death	[73]
Histoplasmosis	Disseminated	*Histoplasma capsulatum*	Nd	Histology	Nd	Recovered	[73]
Histoplasmosis	Disseminated	*Histoplasma capsulatum*	Nd	Histology	Nd	Death	[112]
Histoplasmosis	Hepatosplenic localizations	*Histoplasma capsulatum*	Nd	Histology	Nd	Death	[112]
Histoplasmosis	Oral lesions	*Histoplasma capsulatum*	No	Positive culture of oral biopsy	Potassium iodide, then N-methyl glucamine antimoniate	Recovered	[113]
Histoplasmosis	Primary pulmonary	*Histoplasma capsulatum*	Nd	Histology	Nd	Improvement	[114]
Histoplasmosis	Disseminated	*Histoplasma capsulatum*	Nd	Histology	Nd	Death	[115]
Histoplasmosis	Primary pulmonary	*Histoplasma capsulatum*	Nd	Histology	Nd	Recovered	[116]
Histoplasmosis	Skin involvement; hepatomegaly	*Histoplasma capsulatum*	No	Histology	Nd	Recovered	[117]
Histoplasmosis	Laryngeal lesions	*Histoplasma capsulatum*	No	Positive culture of oral biopsy; histology	N-methyl glucamine antimoniate, nystatin, sulfonamides	Recovered	[118]
Histoplasmosis	Disseminated	*Histoplasma capsulatum*	Nd	Positive culture of blood and bone marrow	Amphotericin B	Death	[98]
Histoplasmosis	Disseminated	*Histoplasma capsulatum*	Nd	Positive culture of bone marrow	Nd	Death	[98]
Histoplasmosis	Acute pulmonary disease	*Histoplasma capsulatum*	Nd	Nd	Nd	Nd	[119]
Histoplasmosis	Disseminated (Lungs; skin; lymph nodes; oropharyngeal and gingival ulcers)	*Histoplasma capsulatum*	No	Histology	Amphotericin B	Recovered	[120]
Histoplasmosis	Disseminated (Lungs; liver)	*Histoplasma capsulatum*	Disseminated candidiasis, myocardial and cerebral toxoplasmosis	Histology	Nd	Nd	[121]
Histoplasmosis	Acute pulmonary disease (mild) with hepatosplenomegaly	*Histoplasma capsulatum*	No	Nd	Nd	Recovered	[122]
Histoplasmosis	Chronic nodular pneumonia	*Histoplasma capsulatum*	No	Histology	Nd	Nd	[94]
Histoplasmosis	Recurrent pulmonary histoplasmosis with hepatosplenomegaly	*Histoplasma capsulatum*	No	Positive culture of lung biopsy; histology; serology	Ketoconazole, then amphotericin B	Recovered	[123]
Histoplasmosis	Pneumonia (mild)	*Histoplasma capsulatum*	No	Histology	Fluconazole	Recovered	[124]
Histoplasmosis	Disseminated (Liver; spleen; lymph nodes; urine)	*Histoplasma capsulatum var capsulatum*	No	Positive culture of urine and lymph node biopsy; histology	Amphotericin B, then Ketoconazole	Recovered	[125]
Histoplasmosis	Tracheobronchial and pulmonary histoplasmosis with cavitation	*Histoplasma capsulatum*	CMV gastroenteritis	Histology	Amphotericin B + ketoconazole	Death	[81]
Histoplasmosis	Disseminated (Lungs; spleen; liver; oropharyngeal, epiglottic and laryngeal ulcers)	*Histoplasma capsulatum*	No	Histology; serology	Amphotericin B, then itraconazole	Recovered	[81]
Histoplasmosis	Disseminated (Lungs; liver; spleen; lymph nodes; bone marrow;)	*Histoplasma capsulatum var capsulatum*	Oropharyngeal candidiasis, visceral leishmaniasis, pulmonary tuberculosis, *S. epidermidis* bacteremia	Positive culture of blood; histology	Fluconazole	Death	[126]
Histoplasmosis	Disseminated (Lungs; skin; bone marrow)	*Histoplasma capsulatum var capsulatum*	Nd	Positive culture of sputum	Amphotericin B	Recovered	[127]
Histoplasmosis	Disseminated (Lungs; spleen; liver; skin; blood; bone marrow)	*Histoplasma capsulatum*	Oropharyngeal candidiasis	Positive culture of blood; histology	Fluconazole	Death	[128]
Histoplasmosis	Disseminated (CNS; spleen; liver; lymph nodes; oropharynx; blood)	*Histoplasma capsulatum var capsulatum*	No	Positive culture of CSF; blood and bone marrow; histology	Amphotericin B, then itraconazole	Death	[129]
Histoplasmosis	Disseminated (skin; bone marrow; lungs; spleen; liver; lymph nodes)	*Histoplasma capsulatum*	No	Histology	Amphotericin B + itraconazole	Death	[130]
Histoplasmosis	Acute pulmonary disease (mild) with hepatosplenomegaly	*Histoplasma capsulatum*	No	Serology	Ketoconazole	Recovered	[104]
Histoplasmosis	Acute pulmonary disease (mild) with hepatosplenomegaly	*Histoplasma capsulatum*	No	Serology	Ketoconazole	Recovered	[104]
Histoplasmosis	Acute pulmonary disease (mild) with hepatosplenomegaly	*Histoplasma capsulatum*	No	Serology	Ketoconazole	Recovered	[104]
Histoplasmosis	Acute pulmonary disease (mild) with hepatosplenomegaly	*Histoplasma capsulatum*	No	Serology	Ketoconazole	Recovered	[105]
Histoplasmosis	Disseminated (skin; blood; liver)	*Histoplasma capsulatum*	No	Positive culture of blood and skin; histology	Fluconazole, then itraconazole	Recovered (died 8 months later for other reasons)	[131]
Histoplasmosis	Chronic pulmonary disease	*Histoplasma capsulatum*	No	Histology	Itraconazole	Recovered	[132]
Histoplasmosis	Disseminated (Heart; blood)	*Histoplasma capsulatum*	Oral candidiasis	Positive culture of blood	Fluconazole	Death	[133]
Histoplasmosis	Disseminated	*Histoplasma capsulatum*	Nd	Nd	Nd	Nd	[134]
Histoplasmosis	Disseminated (Bone marrow; skin; liver; lymph nodes; spleen; blood)	*Histoplasma capsulatum*	Lue	Positive culture of blood, skin and bone marrow; histology	Amphotericin B, then itraconazole	Recovered	[135]
Histoplasmosis	Disseminated (Lungs; bone marrow; liver; lymph nodes; spleen; kidney; heart)	*Histoplasma capsulatum*	No	Histology (post mortem)	None	Death	[135]
Histoplasmosis	Disseminated (Lungs; skin; blood)	*Histoplasma capsulatum var capsulatum*	No	Positive culture of blood; histology	Amphotericin B	Recovered (died 1 year later for other opportunistic infections)	[8]
Histoplasmosis	Acute pulmonary disease	*Histoplasma capsulatum var capsulatum*	No	Histology	Itraconazole	Recovered	[8]
Histoplasmosis	Disseminated (Lungs; blood)	*Histoplasma capsulatum var capsulatum*	No	Positive cultures of blood; serology	Itraconazole	Recovered (died 2 years later for Kaposi Sarcoma)	[8]
Histoplasmosis	Acute pulmonary disease (mild) with hepatosplenomegaly	*Histoplasma capsulatum var capsulatum*	No	Serology	Ketoconazole	Recovered	[8]
Histoplasmosis	Disseminated (Lungs; skin; blood)	*Histoplasma capsulatum var capsulatum*	No	Positive cultures of blood; histology	Amphotericin B	Nd	[8]
Histoplasmosis	Acute pulmonary disease	*Histoplasma capsulatum var capsulatum*	No	Serology	Ketoconazole, then itraconazole	Recovered	[8]
Histoplasmosis	Acute pulmonary disease	*Histoplasma capsulatum var capsulatum*	No	Serology	Amphotericin B, then itraconazole	Recovered	[8]
Histoplasmosis	Large hand ulcer	*Histoplasma capsulatum*	No	Positive culture of skin biopsy; histology	Itraconazole, then fluconazole	Recovered	[100]
Histoplasmosis	Adrenal insufficiency (of bilateral surrenal masses)	*Histoplasma capsulatum var capsulatum*	*Helicobacter pylori* gastritis	Histology	Itraconazole	Recovered	[103]
Histoplasmosis	Ulcerated lesion of the tongue	*Histoplasma capsulatum*	No	Histology	Fluconazole	Recovered	[136]
Histoplasmosis	Pulmonary histoplasmosis with nodules	*Histoplasma capsulatum*	Nd	Nd	Nd	Nd	[137]
Histoplasmosis	Pulmonary histoplasmosis with focal consolidation	*Histoplasma capsulatum*	Nd	Nd	Nd	Nd	[137]
Histoplasmosis	Disseminated (Skin; Lungs)	*Histoplasma capsulatum var capsulatum*	No	Histology; PCR	Nd	Lost to follow up	[107]
Histoplasmosis	Disseminated (Lungs with mediastinal lymph node involvement; blood; skin; spleen)	*Histoplasma capsulatum*	Bacterial pneumonia	Positive culture of blood; histology	Amphotericin B, then itraconazole	Recovered	[138]
Histoplasmosis	Papular-ulcerative lesions of trunk, arms and face	*Histoplasma capsulatum var capsulatum*	No	Positive culture of skin biopsy; histology; PCR	Itraconazole	Recovered	[139]
Histoplasmosis	Papular-ulcerative lesions of trunk, arms and face	*Histoplasma capsulatum var capsulatum*	Esophageal candidiasis	Histology	Itraconazole	Recovered	[140]
Histoplasmosis	Disseminated (Lungs; spleen; liver; lymph nodes; skin; ulcerated palatal lesion)	*Histoplasma capsulatum var capsulatum*	Oropharyngeal candidiasis	Positive culture os skin biopsy; histology; serology	Fluconazole, then itraconazole	Recovered	[140]
Histoplasmosis	Disseminated (Spleen; blood; lymph nodes)	*Histoplasma capsulatum*	No	Positive culture of blood; histology	Itraconazole	Nd	[141]
Histoplasmosis	Acute pulmonary disease	*Histoplasma capsulatum var capsulatum*	No	Serology	Itraconazole	Recovered	[7]
Histoplasmosis	Disseminated (Lungs; blood)	*Histoplasma capsulatum var capsulatum*	Salmonellosis	Positive cultures of blood; histology	Amphotericin B, then itraconazole	Recovered	[7]
Histoplasmosis	Disseminated (Lungs; lymph nodes; blood)	*Histoplasma capsulatum var capsulatum*	PJP	Positive cultures of blood	Itraconazole	Death	[7]
Histoplasmosis	Disseminated (Lungs; blood)	*Histoplasma capsulatum var capsulatum*	PJP, vaginal and tracheo-bronchial candidiasis	Positive cultures of blood	Amphotericin B, then itraconazole	Recovered	[7]
Histoplasmosis	Disseminated (Liver; spleen; lymph nodes; bone marrow)	*Histoplasma capsulatum var duboisii*	Mac	Histology	Itraconazole	Recovered	[6]
Histoplasmosis	Disseminated (Skin; liver; lungs; spleen; lymph nodes; kidney; heart; brain; stomach; uterus; ovary; adrenal glands)	*Histoplasma capsulatum var capsulatum*	PJP	Histology (post mortem)	None	Death	[6]
Histoplasmosis	Disseminated (Skin; lungs; liver; lymph nodes)	*Histoplasma capsulatum var capsulatum*	No	Histology (post mortem)	None	Death	[6]
Histoplasmosis	Disseminated (Lungs; skin; blood)	*Histoplasma capsulatum var capsulatum*	No	Positive culture of blood and skin; histology	Amphotericin B, then itraconazole	Lost to follow up	[6]
Histoplasmosis	Solitary Pulmonary Nodule	*Histoplasma capsulatum*	No	Histology	Surgery	Recovered	[99]
Histoplasmosis	Disseminated (Lungs; spleen; lymph nodes)	*Histoplasma capsulatum var capsulatum*	No	Histology	Amphotericin B	Recovered	[95]
Histoplasmosis	Disseminated (Skin; bone marrow; spleen; liver; lymph nodes)	*Histoplasma capsulatum var capsulatum*	*Strongyloides stercoralis* infection	Histology	Amphotericin B, then itraconazole	Lost to follow up	[142]
Histoplasmosis	Disseminated (Lungs; palatal ulcer; spleen; liver; lymph nodes)	*Histoplasma capsulatum var capsulatum*	Pulmonary tuberculosis	Positive culture of palatal biopsy; histology	Amphotericin B, then fluconazole	Lost to follow up	[142]
Histoplasmosis	Disseminated (Lungs; bone marrow; skin)	*Histoplasma capsulatum var capsulatum*	*Strongyloides stercoralis* infection, oral candidiasis	Histology	Amphotericin B, then itraconazole	Recovered	[142]
Histoplasmosis	Disseminated (Kidney; bone marrow; lymph nodes; spleen; liver)	*Histoplasma capsulatum var capsulatum*	Disseminated CMV	Positive culture of bone marrow; histology	Amphotericin B, then itraconazole	Recovered	[142]
Histoplasmosis	Ulcerated palatal lesion	*Histoplasma capsulatum*	No	Histology	Nd	Nd	[143]
Histoplasmosis	Disseminated (Lungs; lymph nodes; CNS; skin; spleen; liver)	*Histoplasma capsulatum*	No	Histology	Amphotericin B, then itraconazole	Recovered	[144]
Histoplasmosis	Endophthalmitis with CNS involvement	*Histoplasma capsulatum*	No	Positive culture of vitreous biopsy; histology; PCR	Fluconazole, then Itraconazole, then Amphotericin B	Death	[102]
Histoplasmosis	Acute pulmonary disease (mild) with involvement of mediastinal lymph nodes, erythema nodosum and polyarthralgia	*Histoplasma capsulatum*	No	Histology; serology	None	Recovered	[145]
Histoplasmosis	Disseminated (Lungs; mediastinal lymph nodes; bone marrow)	*Histoplasma capsulatum var capsulatum*	No	Positive culture of BAL and bone marrow; PCR	Caspofungin, then Amphotericin B	Death	[101]
Histoplasmosis	Disseminated (Lymph nodes; spleen; liver)	*Histoplasma capsulatum*	Cerebral toxoplasmosis	Positive culture of lymph nodes; histology; serology	Amphotericin B, then itraconazole	Recovered	[97]
Histoplasmosis	Disseminated (Lungs; lymph nodes)	*Histoplasma capsulatum*	Esophageal candidiasis	PCR	Amphotericin B, then itraconazole	Recovered	[97]
Histoplasmosis	Disseminated (Lungs; skin)	*Histoplasma capsulatum var capsulatum*	No	Positive culture of skin and lungs biopsy; histology; positive β-D-glucan and galactomannan	Amphotericin B, then itraconazole	Recovered	[93]
Histoplasmosis	Disseminated (Lungs; liver; kidney; lymph nodes)	*Histoplasma capsulatum*	Disseminated candidiasis	Positive culture of blood; histology (post mortem)	Nd	Death	[146]
Histoplasmosis	Disseminated (Bone marrow; lymph nodes; tonsils; gastro-intestinal)	*Histoplasma capsulatum*	No	Positive culture of bone marrow; histology	Amphotericin B, then itraconazole	Recovered	[85]
Histoplasmosis	Disseminated (Lungs; skin; spleen; liver; lymph nodes)	*Histoplasma capsulatum*	No	Positive culture of blood and bone marrow; histology	Amphotericin B, then itraconazole	Recovered	[96]
Histoplasmosis	Acute pulmonary disease; 1/17 Disseminated	*Histoplasma capsulatum var capsulatum*	No	Serology (2/15)	Itraconazole (7/17)	Recovered	[92]
Histoplasmosis	Acute pulmonary disease	*Histoplasma capsulatum var capsulatum*	No	Nd	Itraconazole	Recovered	[92]
Histoplasmosis	Acute pulmonary disease	*Histoplasma capsulatum var capsulatum*	No	Nd	Itraconazole	Recovered	[92]
Histoplasmosis	Acute pulmonary disease	*Histoplasma capsulatum var capsulatum*	No	Nd	Itraconazole	Recovered	[92]
Histoplasmosis	Acute pulmonary disease	*Histoplasma capsulatum var capsulatum*	No	Nd	Itraconazole	Recovered	[92]
Histoplasmosis	Acute pulmonary disease	*Histoplasma capsulatum var capsulatum*	No	Positive culture of BAL; serology	Itraconazole	Recovered	[92]
Histoplasmosis	Disseminated (Lungs; bone marrow; lymph nodes)	*Histoplasma capsulatum var capsulatum*	No	Positive culture of BAL; histology; serology	Itraconazole	Recovered	[92]
Histoplasmosis	Disseminated (Lungs; lymph nodes; blood; gastrointestinal)	*Histoplasma capsulatum*	No	Microscopic smear identification; positiver culture of blood; serology	Amphotericin B	Death	[147]
Histoplasmosis	Disseminated (Lungs; liver; spleen; lymph nodes)	*Histoplasma capsulatum*	Oral candidiasis	Histology; PCR	Amphotericin B, then itraconazole	Recovered	[4]

*CMV* Cytomegalovirus, *HBV* Hepatitis B virus, *HCV* Hepatitis C virus, *IDU* Intravenous drug user, *PJP Pneumocystis jirovecii* pneumonia, *BAL* Bronchoalveolar lavage

## Discussion

**Fig. 4:**
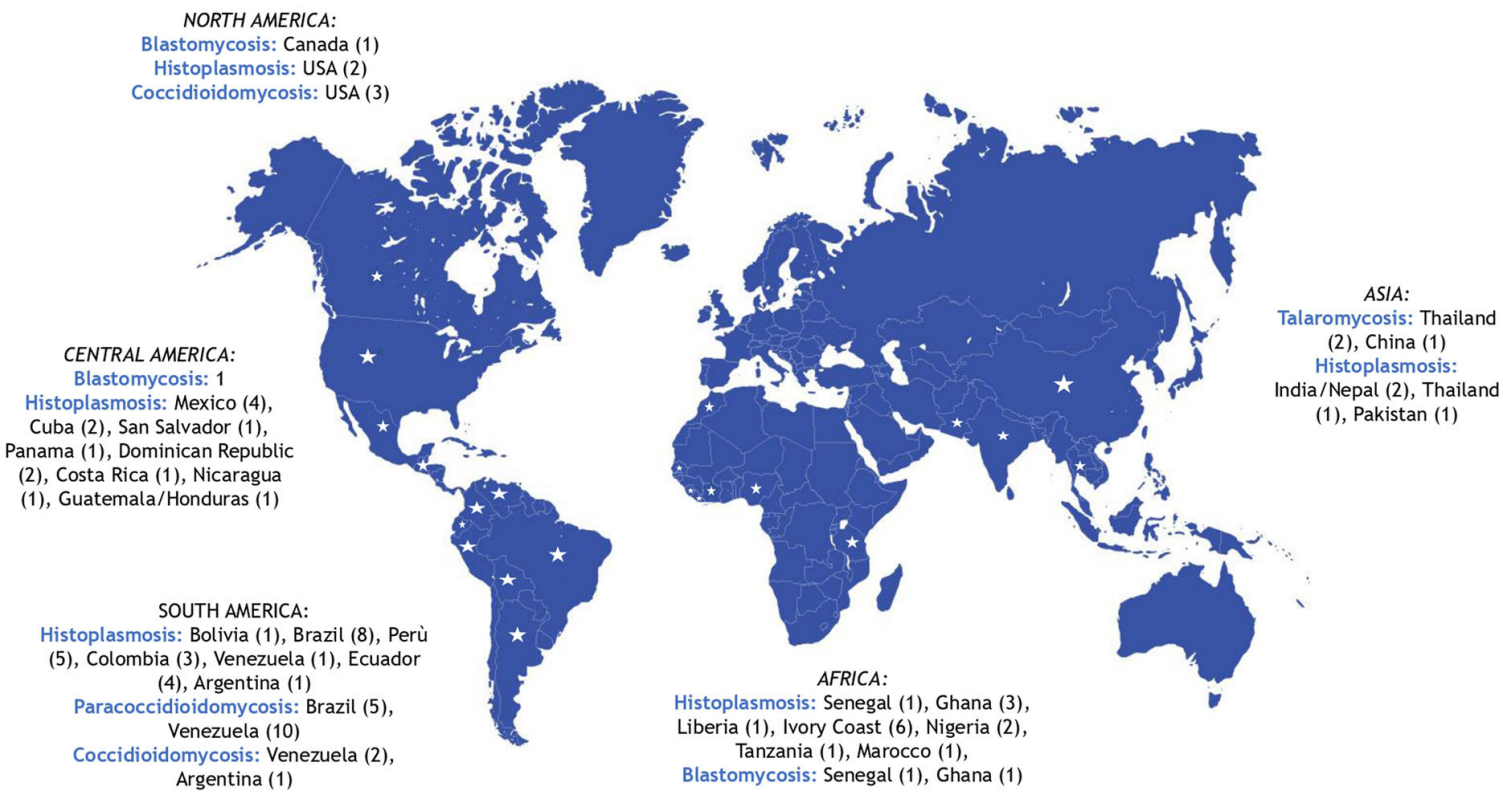
Countries of infection acquisition

We found out from 1914 to nowadays: 105 cases of histoplasmosis, 15 of paracoccidioidomycosis, 10 of coccidioidomycosis, 10 of blastomycosis and 3 of talaromycosis reported in Italy (Fig. 4). The most reported infection in Italy is histoplasmosis and this probably reflects its global distribution [79]. Typically, cases of endemic mycoses in non endemic countries are described in travelers, expatriates and migrants [1]. The understanding of the epidemiology of such diseases is still in progress [105].

In Italy a national surveillance system of endemic mycoses is missing. Moreover, the diagnosis of these mycoses is often difficult because clinical experience of physicians and diagnostic tests both are lacking (except for some reference centers). Thus, our data probably don’t reflect the real epidemiology of endemic mycoses in the country. In Spain, recently, Molina-Morant et al. reviewed the literature about endemic mycoses in the country between 1997 and 2014. There were 286 cases of histoplasmosis, 94 of coccidioidomycosis and 25 of paracoccidioidomycosis [106].

The period between the last time spent in an endemic area and the time of diagnosis sometimes is very long and could last years [62, 74, 107]. This latency period usually is longer for migrants or expatriates rather than travelers [4]. This reminds us how important it is to accurately collect the entire traveling history of the patient.

Among 143 cases of fungal infections reported here, death occurs in 21 cases (20 histoplasmosis, and one paracoccidioidomycosis). Mortality rate was 14.7% (21 deaths/143 cases). Immunosuppression is a major risk factor for getting these mycoses and for more severe outcomes [8]. Immunocompromised patients were 54 and among them 46 had HIV/AIDS. Mortality rate was 26% in immunocompromised patients (14 deaths/54 cases). Other reported conditions of immunosuppression were: cancer, inflammatory bowel diseases, sarcoidosis, rheumatologic disorders. We collect only one case of histoplasmosis in a lung transplant recipient [101]. However, this topic is emerging worldwide, considering the increasing use of immunosuppressive drugs for many diseases. In 2019 the American Society of Transplantation published their guidelines on diagnosis, prevention and management of blastomycosis, histoplasmosis, and coccidioidomycosis, that are endemic in USA, in the pre‐ and post- transplant period [108].

For histoplasmosis (n = 28), coccidioidomycosis (n = 4), and blastomycosis (n = 6), cases in people who never traveled or have links to endemic regions have been reported. So, these cases could be considered as autochthonous. In particular, evidence about the presence of *Histoplasma* spp in Italy has been documented by isolation in soil [109], cases in animals [110, 111] and histoplasmin reactivity surveys [81, 98]. No similar studies have been conducted in Italy for coccidioidomycosis, and blastomycosis. Nevertheless, species identification for the above mentioned isolations from soil or animals only relied on morphological features, and only one of these possible autochthonous cases was diagnosed by PCR-based tests.

In non-endemic countries the diagnosis of endemic mycoses could be challenging. Even if the culture remains the gold standard, PCR based tests detected the fungus in 8 cases (4 of these with negative cultures of clinical samples). Most of the described cases were diagnosed by histology and/or cultures. Serology and antigen testing have been less diriment.

## Conclusions

The increasing trend of international travels, migration flows alongside the increasing number of persons living with immunosuppression conditions (e.g. solid organ transplants) has led to an increase of imported cases of endemic mycoses in non endemic countries such as Italy. A story of travels and immunosuppression should lead clinicians to consider endemic mycoses in differential diagnosis of systemic diseases.

## Supplementary Information

Below is the link to the electronic supplementary material.

Supplementary file1 (DOCX 16 KB)
