# Supplementary material for: Endemic Systemic Mycoses in Italy: A Systematic Review of Literature and a Practical Update
Source: Mycopathologia. 2023 Jun 9;188(4):307–34. doi: 10.1007/s11046-023-00735-z (PMC10386973; doi:10.1007/s11046-023-00735-z)
Supplement: Supplementary file 1 — Supplementary file1 (DOCX 16 KB) [file 11046_2023_735_MOESM1_ESM.docx]

***SEARCH STRATEGY***

**MEDLINE (PubMed) (1966 to April 2022)**

((("Mycoses"[MeSH Terms] OR "Histoplasma"[MeSH Terms] OR "Coccidioidomycosis"[MeSH Terms] OR "Blastomyces"[MeSH Terms] OR ("mycos*"[Title/Abstract] OR "fungus disease*"[Title/Abstract] OR "fungus infection*"[Title/Abstract] OR "fungal infections*"[Title/Abstract] OR "fungal disease*"[Title/Abstract] OR "fung*"[Title/Abstract] OR "Talaromyces marneffei"[Title/Abstract] OR "talaromyc*"[Title/Abstract] OR "Paracoccidioides*"[Title/Abstract] OR "paracoccidioidomyc*"[Title/Abstract] OR "histoplasm*"[Title/Abstract] OR "histoplasma*"[Title/Abstract] OR "Histoplasma capsulatum"[Title/Abstract] OR "Emmonsiella capsulata"[Title/Abstract] OR "Ajellomyces capsulatus"[Title/Abstract] OR "coccidioidomycos*"[Title/Abstract] OR "coccidioides immitis infection*"[Title/Abstract] OR "San Joaquin Valley Fever"[Title/Abstract] OR "valley fever*"[Title/Abstract] OR "blastomyc*"[Title/Abstract] OR "Blastomyces dermatitidis"[Title/Abstract] OR "Ajellomyces dermatitidis"[Title/Abstract]))) AND (("Travel"[MeSH Terms] OR "Refugees"[MeSH Terms] OR ("Emigrants and Immigrants"[MeSH Terms] OR "Transients and Migrants"[MeSH Terms]) OR ("Refugees"[Title/Abstract] OR "asylum seekers"[Title/Abstract] OR "migrant*"[Title/Abstract] OR "transient*"[Title/Abstract] OR "squatter*"[Title/Abstract] OR "travel*"[Title/Abstract] OR "migrant worker*"[Title/Abstract] OR "nomad*"[Title/Abstract] OR "acquir*"[Title/Abstract] OR "vacat*"[Title/Abstract] OR "holiday*"[Title/Abstract] OR "expatriates"[Title/Abstract])))) AND (("Italy"[Mesh]) OR (Italy[Title/Abstract] OR Italia[Title/Abstract]))

**Embase (Embase.com) (1974 to April 2022)**

#1 'mycosis'/exp/mj OR 'histoplasma'/exp/mj OR 'coccidioidomycosis'/exp/mj OR 'blastomyces'/exp/mj

#2 mycos* OR fungus OR 'fungus disease*' OR 'fungus infection*' OR 'fungal infections*' OR 'fungal disease*' OR fung* OR histoplasm* OR histoplasma* OR 'histoplasma capsulatum' OR 'emmonsiella capsulata' OR 'ajellomyces capsulatus' OR 'cryptococcus capsulatus' OR coccidioidomycos* OR 'coccidioides immitis infection*' OR 'san joaquin valley fever' OR 'valley fever*' OR blastomyc* OR 'blastomyces dermatitidis' OR 'blastomycoides dermatitidis' OR 'ajellomyces dermatitidis' OR Talaromyces marneffei OR 'talaromyc*' OR 'Paracoccidioides' OR paracoccidioidomyc*':ti,ab

#3 #1 OR #2

#4 'travel'/exp/mj OR 'refugee'/exp/mj OR 'migrant'/exp/mj OR 'migration'/exp/mj

#5 refugees OR 'asylum seekers' OR migrant* OR transient* OR squatter* OR travel* OR 'migrant worker*' OR nomad* OR acquir* OR vacat* OR holiday* OR expatriates:ti,ab

#6 #4 OR #5

#7 'italy'/exp/mj

#8 'italy' OR italia:ti,ab

#9 #7 OR #8

#10 #3 AND #6 AND #9

#11 #3 AND #6 AND #9 AND [embase]/lim

**Cochrane Central Register of Controlled Trials (CENTRAL; 2021, Issue 4) in the Cochrane Library (searched 01 April 2022)**

#1 MeSH descriptor: [Mycoses] explode all trees

#2 MeSH descriptor: [Histoplasma] explode all trees

#3 MeSH descriptor: [Coccidioidomycosis] explode all trees

#4 #1 OR #2 OR #3

#5 (mycos* OR fungus OR “Fungus Disease*” OR “Fungus Infection*” OR “Fungal Infections*” OR “Fungal Disease*” OR fung* OR “Talaromyces marneffei*” OR Paracoccidioides OR talaromyc* OR paracoccidioidomyc* OR Histoplasm* OR Histoplasma* OR “Histoplasma capsulatum” OR “Emmonsiella capsulata” OR “Ajellomyces capsulatus” OR “Cryptococcus capsulatus” OR Coccidioidomycos* OR “Coccidioides immitis Infection*” OR “San Joaquin Valley Fever” OR “Valley Fever*” OR Blastomyc* OR “Blastomyces dermatitidis” OR “Blastomycoides dermatitidis” OR “Ajellomyces dermatitidis”:ti,ab,kw

#6 #4 OR #5

#7 MeSH descriptor: [Italy] explode all trees

#8 (Italy OR Italia):ti,ab,kw

#9 #7 OR #8

#10 #6 AND #9

#11 MeSH descriptor: [Travel] explode all trees

#12 MeSH descriptor: [Refugees] explode all trees

#13 MeSH descriptor: [Emigrants and Immigrants] explode all trees

#14 MeSH descriptor: [Transients and Migrants] explode all trees

#15 (refugees OR "asylum seekers" OR Migrant* OR Transient* OR Squatter* OR travel* OR "Migrant Worker*" OR Nomad* OR acquir* OR vacat* OR holiday* OR expatriates):ti,ab,kw

#16 #11 OR #12 OR #13 OR #14 OR #15

#17 #6 AND #10 AND #16
